# Oncostatin M-driven macrophage-fibroblast circuits as a drug target in autoimmune arthritis

**DOI:** 10.1186/s41232-024-00347-0

**Published:** 2024-07-31

**Authors:** Nam Cong-Nhat Huynh, Rui Ling, Masatsugu Komagamine, Tianshu Shi, Masayuki Tsukasaki, Kotaro Matsuda, Kazuo Okamoto, Tatsuo Asano, Ryunosuke Muro, Warunee Pluemsakunthai, George Kollias, Yuko Kaneko, Tsutomu Takeuchi, Sakae Tanaka, Noriko Komatsu, Hiroshi Takayanagi

**Affiliations:** 1 Department of Immunology, Graduate School of Medicine and Faculty of Medicine, The University of Tokyo, Tokyo, Japan; 2 Unit of Prosthodontics, Faculty of Odonto-Stomatology, University of Medicine and Pharmacy at Ho Chi Minh City, Ho Chi Minh City, Vietnam; 3 Division of Rheumatology, Department of Internal Medicine, Keio University School of Medicine, Tokyo, Japan; 4 Department of Osteoimmunology, Graduate School of Medicine and Faculty of Medicine, The University of Tokyo, Tokyo, Japan; 5 Division of Immune Environment Dynamics, Cancer Research Institute of Kanazawa University, Kakuma-Machi, Kanazawa, Japan; 6 Institute for Bioinnovation, Biomedical Sciences Research Center (BSRC), Alexander Fleming’, Vari, Attika, Greece; 7 Department of Physiology, Medical School, National and Kapodistrian University of Athens, Athens, Greece; 8 Saitama Medical University, Saitama, Japan; 9 Department of Orthopaedic Surgery, Faculty of Medicine, The University of Tokyo, Tokyo, Japan; 10 Department of Immune Regulation, Medical Research Institute, Tokyo Medical and Dental University (TMDU), Tokyo, Japan

**Keywords:** Rheumatoid arthritis, JAK inhibitor, Macrophage, Synovial fibroblast, Drug target

## Abstract

**Background:**

Recent single-cell RNA sequencing (scRNA-seq) analysis revealed the functional heterogeneity and pathogenic cell subsets in immune cells, synovial fibroblasts and bone cells in rheumatoid arthritis (RA). JAK inhibitors which ameliorate joint inflammation and bone destruction in RA, suppress the activation of various types of cells in vitro. However, the key cellular and molecular mechanisms underlying the potent clinical effects of JAK inhibitors on RA remain to be determined. Our aim is to identify a therapeutic target for JAK inhibitors in vivo*.*

**Methods:**

We performed scRNA-seq analysis of the synovium of collagen-induced arthritis (CIA) mice treated with or without a JAK inhibitor, followed by a computational analysis to identify the drug target cells and signaling pathways. We utilized integrated human RA scRNA-seq datasets and genetically modified mice administered with the JAK inhibitor for the confirmation of our findings.

**Results:**

scRNA-seq analysis revealed that oncostatin M (OSM) driven macrophage-fibroblast interaction is highly activated under arthritic conditions. OSM derived from macrophages, acts on OSM receptor (OSMR)-expressing synovial fibroblasts, activating both inflammatory and tissue-destructive subsets. Inflammatory synovial fibroblasts stimulate macrophages, mainly through IL-6, to exacerbate inflammation. Tissue-destructive synovial fibroblasts promote osteoclast differentiation by producing RANKL to accelerate bone destruction. scRNA-seq analysis also revealed that OSM-signaling in synovial fibroblasts is the main signaling pathway targeted by JAK inhibitors in vivo. Mice specifically lacking OSMR in synovial fibroblasts (*Osmr*^∆Fibro^) displayed ameliorated inflammation and joint destruction in arthritis. The JAK inhibitor was effective on the arthritis of the control mice while it had no effect on the arthritis of *Osmr*^∆Fibro^ mice.

**Conclusions:**

OSM functions as one of the key cytokines mediating pathogenic macrophage-fibroblast interaction. OSM-signaling in synovial fibroblasts is one of the main signaling pathways targeted by JAK inhibitors in vivo. The critical role of fibroblast-OSM signaling in autoimmune arthritis was shown by a combination of mice specifically deficient for OSMR in synovial fibroblasts and administration of the JAK inhibitor. Thus, the OSM-driven synovial macrophage-fibroblast circuit is proven to be a key driver of autoimmune arthritis, serving as a crucial drug target in vivo*.*

**Supplementary Information:**

The online version contains supplementary material available at 10.1186/s41232-024-00347-0.

## Background

Rheumatoid arthritis (RA) is an autoimmune disease characterized by joint inflammation and bone destruction [1–3]. In RA, immune cells, including T cells and macrophages, and synovial fibroblasts become activated and proliferate in arthritic synovium, resulting in inflammation and bone destruction in joints [1–5]. Cytokines, interferons, and growth factors are the key mediators of the immune cell-fibroblast-bone triangle interaction that underlies the pathogenesis of RA. Janus kinases (JAKs: JAK1, JAK2, JAK3 and TYK2) are widely expressed in various cells, including immune, stromal and bone cells in joints, and are involved in a variety of cellular responses initiated by these key mediators [6, 7]. JAKs phosphorylate STATs, induce multimerization and translocate them into the nucleus in order to regulate gene transcription. JAK inhibitors suppress joint inflammation and bone destruction to an extent similar to biological DMARDs (bDMARDs) [6, 7]. bDMARDs specifically target the molecule the antibodies recognize. However, it is still unclear which cells and signaling pathways are the main targets of JAK inhibitors in vivo, since most immune, stromal and bone cells are influenced by mediators that utilize the JAK-STAT pathways [6–8]. In vitro studies have shown that JAK inhibitors suppress the activation of T cells, dendritic cells and synovial fibroblasts [9–13]. JAK inhibitors inhibit osteoclastogenesis by inhibiting RANKL expression on osteoblastic cells but not affecting osteoclast precursor cells, while a recent report showed that JAK inhibitors affect migration of osteoclast precursors [14–16]. JAK inhibitors are reported to promote osteoblastic bone formation in vitro, while it was shown that JAK inhibitors ameliorate joint erosion mainly by inhibiting osteoclastogenesis in vivo [15, 17]*. *However, the key cellular and molecular mechanisms underlying the potent clinical effects of JAK inhibitors on RA remain to be determined.

In this study, we performed a scRNA-seq analysis of the synovium of collagen-induced arthritis (CIA) mice treated with or without a JAK inhibitor, upadacitinib, followed by a computational analysis to identify the drug target cells and signaling pathways [17, 18]. We found that the interplay between synovial macrophages and fibroblasts was critically inhibited by the JAK inhibitor treatment. The JAK inhibitor mainly targeted OSM signaling in synovial fibroblasts. We integrated human RA scRNA-seq data sets and revealed the importance of the OSM-mediated macrophage-synovial fibroblast interaction. OSM promoted the expression of both inflammatory and tissue-destructive genes from synovial fibroblasts. These results suggest that OSM is the common activator of inflammatory and tissue-destructive synovial fibroblasts and JAK inhibitors primarily targets the activation of both types of synovial fibroblasts by inhibiting OSM-driven pathways. Inflammatory and tissue-destructive fibroblasts in turn stimulate macrophages through inflammatory cytokines such as IL-6 and RANKL, respectively, thus forming a vicious circle.

Importantly, mice specifically lacking OSM receptors in synovial fibroblasts exhibited ameliorated inflammation and joint destruction in arthritis in vivo. These results illustrate the importance of fibroblast-OSM signaling in the immune cell-fibroblast-bone interaction in arthritis, providing novel insights into the pathogenesis of RA and the key targets of JAK inhibitors.

## Results

### scRNA-seq characterization of synovial fibroblasts and macrophages the most prevalent populations in arthritic synovium

**Fig. 1:**
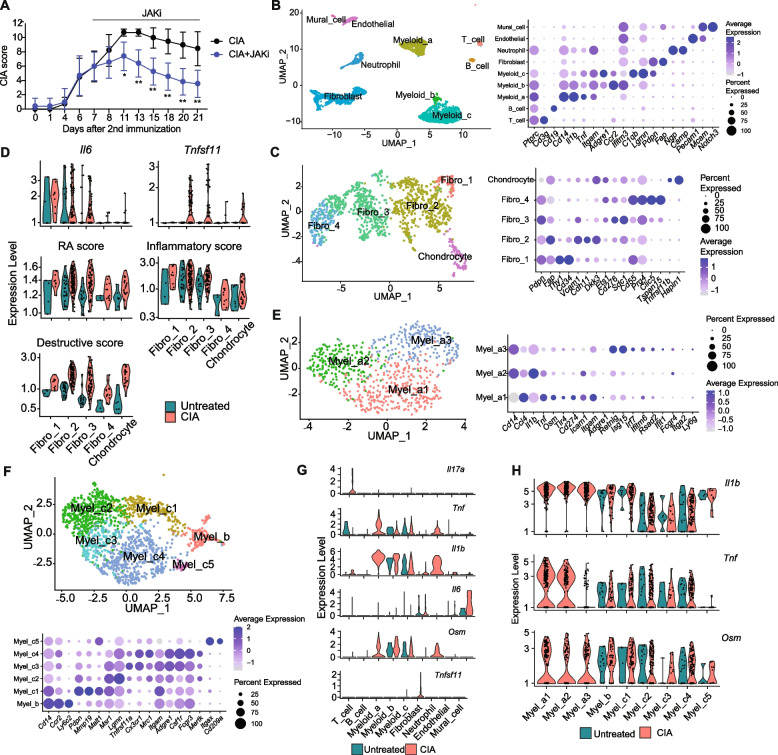
Characterization of synovial fibroblasts and macrophages in CIA synovium by single-cell analysis. **A** The arthritis score of CIA mice which were administered upadacitinib, a JAK inhibitor (JAKi) from 7 to 21 days after the 2nd immunization. **B** Main clusters of the sc-RNAseq data in the untreated, CIA and CIA + JAKi group (left), and the average expression of the representative markers of each cell type (right). **C** Sub-clusters of synovial fibroblasts (left), and the average expression of the representative markers (right). **D** Expression of *Il6* and *Tnfsf11* (top), RA, inflammatory and destructive scores (middle and bottom) in synovial fibroblast. **E** Sub-clusters of *Itgam* (CD11b)^+^ *Adgre1* (F4/80)^−^ circulating monocytes Myel_a (left panel), and the average expression of markers (right panel). **F** Sub-clusters of Itgam^+^Adgre1.^+^ macrophages Myel_b and _c (left) and the average expression of representative markers (right). **G** The expression level of *Il17a, Tnf, Il1b, Il6, Osm,* and *Tnfsf11* genes in main-clusters under the untreated and CIA conditions. **H** The expression level of *Il1b, Tnf,* and *Osm* genes in all myeloid sub-clusters under untreated and CIA conditions. All data are expressed as the mean ± SD with *n* = 4–7, *p < 0.05, **p < 0.01, by unpaired Student's t-test (A)

CIA mice treated with the JAK inhibitor, upadaticinib, after the onset of arthritis exhibited alleviated arthritis scores compared with control CIA mice [17] (Fig. 1A, Fig. S1A). As we focused on the effect of the JAK inhibitor in the bone destruction phase, the JAK inhibitor was administered from day 7 to day 21 after secondary immunization. We analyzed the integrated scRNA­seq data of synovial tissue from untreated mice, CIA mice, and CIA mice treated with the JAK inhibitor (Fig. 1B, Fig. S1B). We identified clusters of T cells, B cells, myeloid cells, neutrophils, and synovial fibroblasts by canonical marker expression (Data file S1) (Fig. 1B). Synovial fibroblasts and myeloid cells were found to be the most abundant cell populations in arthritis, consistent with previous reports [19].

 The synovial fibroblast cluster was further divided into fibro_1 (*Thy1*^hi^*Cd34*^hi^), fibro_2 (*Thy1*^low^*Cdh11*^hi^), fibro_3 (*Thy1*^*−*^* Cd276*^hi^), and fibro_4 (*Thy1*^*−*^* Prg4*^hi^) subpopulations (Fig. 1C, Fig. S1C). In the fibroblast cluster, we detected the chondrocyte population (*Pdpn*^low^) characterized by the expression of the cartilage-related genes, such as *Hapln1*. Based on Thy1 expression, fibro_1 (*Thy1*^hi^) was assigned to the sublining fibroblasts while fibro_2 *(Thy1*^low^) and fibro_3 and _4 (*Thy1*^*−*^) were assigned to lining cells (Fig. 1C) [4, 20, 21]. We evaluated the expression of genes involved in RA pathogenesis in each fibroblast subset. The expression of inflammatory genes such as IL-6 was the highest in fibro_1, while the expression of tissue-destructive genes such as *Tnfsf11* (encoding RANKL), *Mmp3* and *Mmp13* was the highest in fibro_2 and _3, suggesting that fibro_1 represents inflammatory synovial fibroblasts and fibro_2 and _3 represent tissue-destructive synovial fibroblasts (Fig. 1D and Fig. S1, F and G). We recently identified Ets1 promotes RANKL expression in tissue-destructive synovial fibroblasts in arthritis [21]. Ets1 was highly expressed not only in RANKL^+^fibro_2 and 3 but also in RANKL^−^fibro_4. Trajectory analysis shaped the transitional model of synovial fibroblast subpopulations from fibro_1 (sublining) via fibro_2 to fibro_3 and then fibro_4 (lining) (Fig. S1, D and E). It is likely that fibro_4 (*Thy1*^−^*Prg4*^hi^*Ets1*^hi^) is at the final stage of fibroblast transition regarded as post-tissue destructive fibroblasts.

In myeloid cells, we identified monocytes (myel_a) (*Itgam* (CD11b)^+^ *Adgre1* (F4/80)^−^) and macrophage*s* (myel_b and _c) (*Itgam*^+^*Adgre1*^+^*)* (Fig. 1, B). Myel_a consists of myel_a1, 2, 3, all of which are *Il1b*^+^ inflammatory monocytes (Fig. 1E). Myel_b was assigned to *Ccr2*^+^*Mertk*^*−*^ infiltrating macrophages. Myel_c was classified into *Mmp19*^+^*Mertk*^*−*^ infiltrating macrophages (myel_c1), *Mertk*^+^*Trem2*^+^ tissue-resident macrophage (myel_c2, 3, 4) and *H2-Aa*^+^*Itgax*^+^ dendritic cells (myel_c5) (Fig. 1F) [22, 23].

The expression of key cytokines such as *Il17a*, *Tnf*, *Il1b*, *Il6, Osm,* and *Tnfsf11* was upregulated under CIA conditions (Fig. 1G, Fig. S2). We found that certain monocytes (myel_a1, 2) expressed a high level of TNF, while OSM was highly expressed in infiltrating macrophages (myel_b, c1) (Fig. 1H). In addition, *Il17a* was predominantly expressed in T cells, whereas *Il6* was highly expressed in fibro_1 and mural cells (Fig. 1, D and G). *Tnfsf11* (RANKL) was exclusively expressed in fibro_2 and _3 among all the synovial cell subsets 3 weeks after the second immunization in CIA (Fig. 1, D and G).

### Gene expression in synovial fibroblasts and macrophages is affected by the JAK inhibitor in autoimmune arthritis

**Fig. 2:**
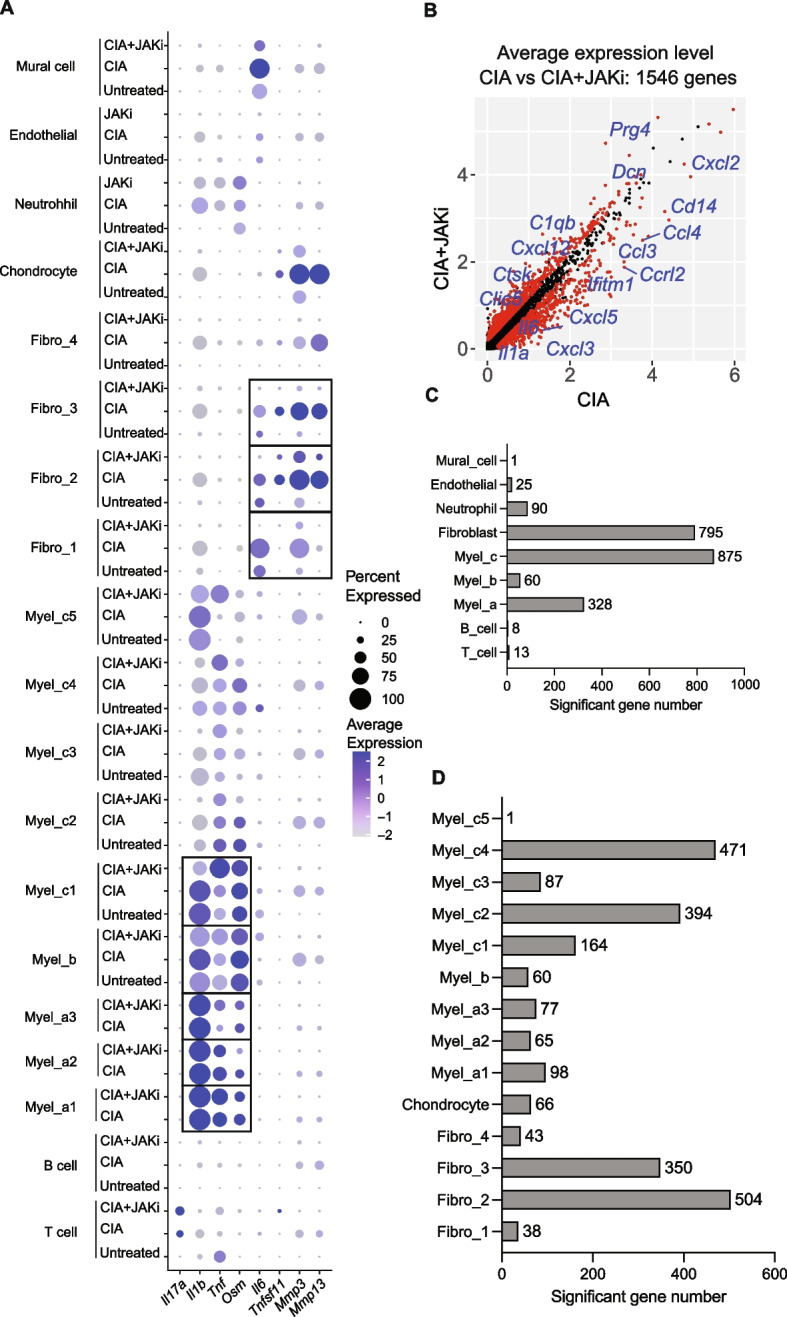
Affected gene expression in synovial fibroblasts and macrophages by the JAK inhibitor in arthritis. **A** Average expression of *Il17a, Il1b, Tnf, Osm, Il6, Tnfsf11, Mmp3* and *Mmp13* across all cell subtypes under untreated, CIA and CIA + JAKi conditions. **B** Average expression of genes in the whole samples (red dots are significantly different genes). **C** Number of genes whose expression was influenced by JAKi in the main clusters under CIA conditions. **D** Number of genes whose expression was influenced by JAKi in the myeloid and synovial fibroblast sub-clusters under CIA conditions

We next compared the gene expression in cells in the synovium under three conditions (untreated, CIA, CIA + the JAK inhibitor). In CIA, the expression of *Il6* [(primarily expressed by, hereafter) inflammatory synovial fibroblasts, fibro_1], as well as *Tnfsf11* and *MMPs* [tissue-destructive synovial fibroblasts, fibro_2 and _3] were increased. The expression of these genes was almost completely abrogated by administration of the JAK inhibitor (Fig. 2A). In contrast, the increased expression of *Tnf* and *Il1β* [myel_a], *Osm* [infiltrating macrophages, myel_b and myel_c1] as well as *Il17a* [T cells] was not reduced by the JAK inhibitor (Fig. 2A).

The expression of 1545 genes was affected by the JAK inhibitor (Fig. 2B, Data file S2). Among all the cell subsets in the synovium, myel_c and synovial fibroblasts had the highest number of genes whose expression was influenced by JAKi (875 genes and 795 genes, respectively) (Fig. 2C, Data file S2). In particular, fibro_2 had the highest number of JAK inhibitor-affected genes (Fig. 2D). Taken together, upon JAK inhibitor treatment, fibroblasts and macrophages were the most affected cells in synovium in terms of gene expression.

### Administration of the JAK inhibitor inhibits the interaction between synovial fibroblasts and macrophages by targeting OSM signaling in synovial fibroblasts in autoimmune arthritis

**Fig. 3:**
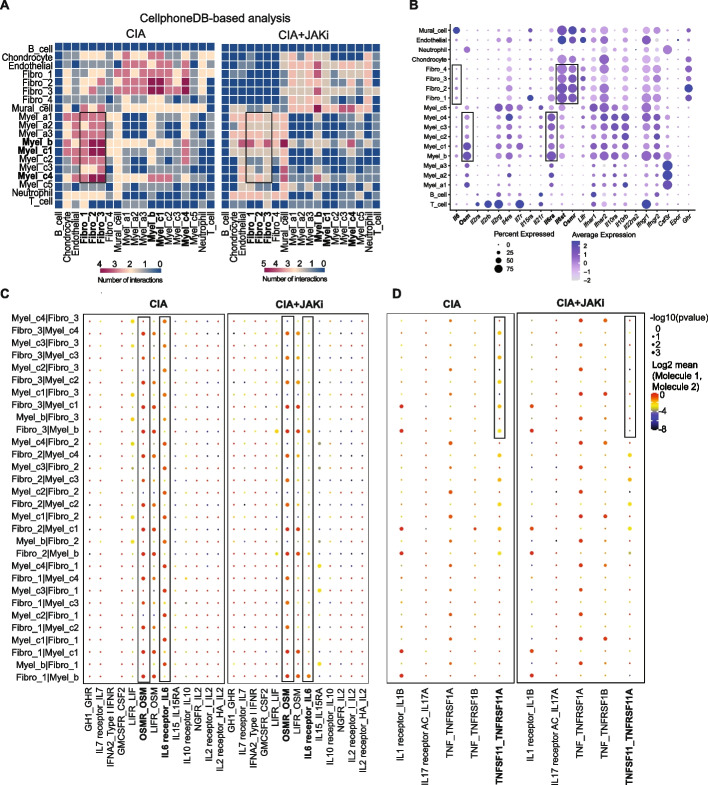
Administration of the JAK inhibitor inhibits the interaction between macrophages and synovial fibroblasts. **A** Cell–cell communication analysis by CellPhoneDB focusing on the expression of JAK/STAT cytokines and receptors: heatmap of cell interactions under CIA (left) and CIA + JAKi (right) conditions. **B** Average expression of JAK/STAT cytokines and receptors in all of the mouse synovial cell subtypes. **C** Dot plots of JAK/STAT cytokines_receptors interaction in myeloid (myel_b and _c1-4) and synovial fibroblast (fibro_1-4) subtypes under CIA (upper) and CIA + JAKi (lower) conditions. **D** Dot plots of IL1B—IL1R, IL17—IL17R A/C, TNF—TNFRSF1A/B, and TNFSF11—TNFRSF11A interactions in myeloid and synovial fibroblast subtypes under CIA (upper) and CIA + JAKi (lower) conditions

To explore the cell–cell interaction that is targeted by the JAK inhibitor, we conducted a CellphoneDB-based analysis focusing on the expression of JAK/STAT cytokines and receptors under CIA and CIA + the JAK inhibitor conditions [24]. Among all the cells in the synovium, the strong JAK/STAT cytokine-receptor interaction was observed between macrophages (especially myel_b and _c1) and synovial fibroblasts (especially fibro_2, 3) under CIA conditions. This interaction was impaired by the JAK inhibitor treatment (Fig. 3A). Among JAK/STAT cytokines and receptors, *Osm* and *Osm receptor* (*Osmr*) were most highly expressed by macrophages and synovial fibroblasts under CIA conditions, respectively (Fig. 3B and C). *Osm* was highly expressed in infiltrating macrophages (myel_b and myel_c1) while OSM receptor components (*Osmr, Il6st*) were predominantly expressed in inflammatory and tissue-destructive synovial fibroblasts (fibro_1-3) (Fig. 3B). OSM is capable of binding to the complex of gp130 and leukemia inhibitory factor receptor (LIFR) at a lower affinity, but the OSMR expression is much higher than LIFR in synovial fibroblasts, suggesting that OSM activates synovial fibroblasts mainly through OSMR under arthritic conditions in mice [25].

The *Osm*/*Osmr* interaction was unaffected by the JAK inhibitor, indicating that JAK inhibitors mainly target OSM signaling in synovial fibroblasts rather than decreasing the expression of OSM or OSMR (Fig. 3C, S3). Since it is reported that OSM upregulates the expression of IL-6 and RANKL in synovial fibroblasts in vitro, we next examined *Il6/Il6r and Tnfsf11/Tnfrsf11a* (encoding RANK) interaction under CIA and CIA + the JAK inhibitor conditions [26, 27]. *Il6* and *Il6r* were expressed by synovial fibroblasts and macrophages under CIA conditions, respectively (Fig. 3, B). Upon the JAK inhibitor administration, the *Il6*/*Il6r* interaction was significantly impaired while the expression of *Il6r* was unaffected, suggesting that JAK inhibitors may suppress the *Il6*/*Il6r* interaction by impairing IL-6 production by synovial fibroblasts in vivo (Fig. 3C, Fig. S3). Likewise, upon the JAK inhibitor administration, the interaction between *TNFSF11* [synovial fibroblasts] and *TNFRSF11A* [macrophages] was impaired while the expression of *TNFRSF11A* was unaffected (Fig. 3D, S3), suggesting that JAK inhibitors may dampen the RANKL/RANK interaction by impairing RANKL expression by synovial fibroblasts in vivo. These results suggest that JAK inhibitors inhibit the macrophage-synovial fibroblast circuit which plays a critical role in inflammation and bone destruction, mainly by targeting OSM signaling in synovial fibroblasts in vivo*.*

**Fig. 4:**
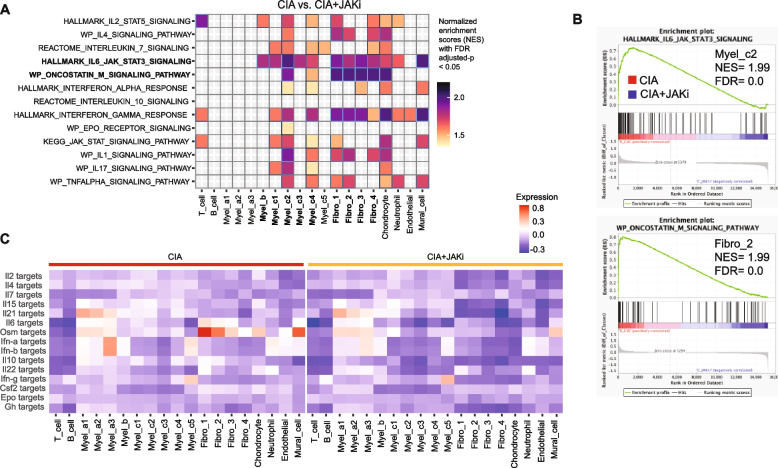
The JAK inhibitor targets the OSM signaling in synovial fibroblasts. **A** Normalized enrichment scores analyzed by GSEA. Heatmap of all clusters in CIA vs. CIA + JAKi conditions using JAK/STAT-related cytokine signaling and RA gene sets with FDR adjusted-*p* < 0.05. **B** The highest NES (GSEA) responses between CIA vs. CIA + JAKi conditions. **C** Heatmaps of the average signature scores of all the mouse synovial cell subtypes. using 15 JAK/STAT pathway cytokine target gene sets under CIA (left) and CIA + JAKI (right) conditions

To gain further insights into the signaling pathway targeted by JAK inhibitors, we performed another unbiased analysis, gene set enrichment analysis (GSEA) which calculates the normalized enrichment scores (NESs) based on gene expression under CIA conditions compared with CIA + the JAK inhibitor conditions. The OSM signaling pathway in CIA synovial fibroblasts marked one of the highest NESs affected by the JAK inhibitor administration, suggesting that JAK inhibitors mainly target the OSM signaling pathway in synovial fibroblasts (Fig. 4A and B). The highest level of NESs was also observed in the IL-6 signaling pathway in CIA macrophages, suggesting impaired expression of IL-6 in synovial fibroblasts may inhibit IL-6-mediated activation of macrophages upon JAKi administration (Fig. 4A and B). These results are consistent with the notion that JAKi mainly targets OSM signaling in synovial fibroblasts in vivo, resulting in impairment of the IL-6 production in synovial fibroblasts and inhibition of IL-6 signaling in macrophages.

To provide further evidence for the importance of synovial fibroblast OSM signaling as a target of JAK inhibitors, we evaluated the expression of genes which are regulated by each JAK/STAT pathway at the single-cell level. We selected a set of 50 genes that are upregulated by each JAK/STAT cytokine based on previously reported microarray or RNA-seq databases, and evaluated the expression of the gene set in all the cell types in the synovium under CIA and CIA + the JAK inhibitor conditions [28–37]. We found that the expression of the OSM-target genes in fibro_1 and 2 was the highest among all the combinations of cytokine-target genes and cell types under CIA conditions, and was completely inhibited by the JAK inhibitor administration (Fig. 4C). We further explored the genes downregulated by the JAK inhibitor administration in synovial fibroblast subsets (Fig. S4). The expression of the OSM-target genes *Ccl2* and *Il6* in inflammatory synovial fibroblasts (fibro_1) as well as *Tnfsf11*, *Mmp3* and *Mmp13* in tissue-destructive fibroblasts (fibro _3) was inhibited by the JAK inhibitor administration (Fig. S4).

Taken together, these results suggest that the macrophage-synovial fibroblast interaction plays a pivotal role in autoimmune arthritis. OSM from macrophages interacts with the OSM receptor on synovial fibroblasts and activates both inflammatory and tissue-destructive synovial fibroblasts. JAK inhibitors mainly target OSM signaling in synovial fibroblasts and thus inhibit the activation of both types of synovial fibroblasts, which in turn inhibits the activation of macrophages, leading to an amelioration of the inflammation and bone destruction in arthritis.

### The OSM-IL-6 circuits underlie the pathogenic interaction between synovial fibroblasts and macrophages in RA

**Fig. 5:**
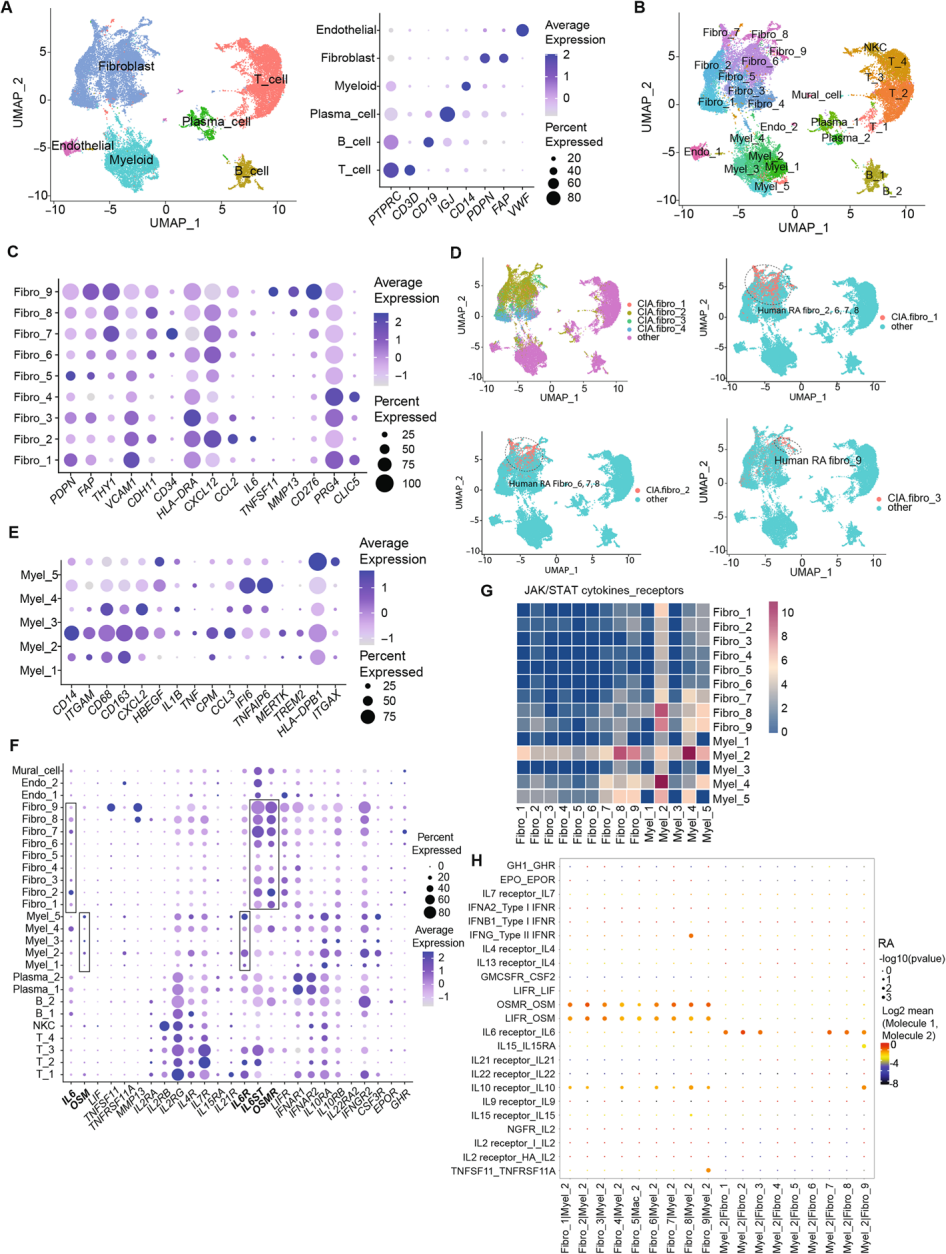
The OSM-driven macrophage-synovial fibroblast circuit in RA synovial cells. **A** Main cell clusters (left) and expression of the representative markers (right) by analysis of the integrated scRNA-seq data of RA synovial cells. **B** Sub-clusters of the integrated RA synovial cells. **C** Expression of representative markers of RA synovial fibroblast sub-clusters. **D** Module scores of mouse CIA fibroblasts markers identified 4 mouse CIA fibroblast subpopulations (upper left) as well as CIA fibro_1 2 and 3 (upper right, lower left and lower right, respectively) in RA synovial cells. **E** Markers of RA synovial myeloid sub-clusters. **F** Average expression of *IL6, OSM, LIF, TNFSF11, TNFRSF11A, MMP13* and JAK/STAT cytokine receptor genes in the RA synovial cell clusters. **G** Heatmaps of JAK/STAT cytokine-receptor interactions in RA synovial myeloid and fibroblast subsets. **H** Dot plots of JAK/STAT cytokines-receptors and RANKL/RANK interactions in RA myel_2 and fibro_1-9

To explore whether a homologous macrophage-synovial fibroblast circuit exists in human autoimmune arthritis, we integrated the previously reported scRNA-seq data on RA synovial tissues and established a novel RA scRNA-seq data set [19, 38]. We obtained 28,158 cells and assigned the main clusters (Fig. 5A and B). Similar to the mouse CIA synovium, *IL1B* and *OSM* were mainly expressed in macrophages, while *IL6* and *TNFSF11* were mainly expressed by synovial fibroblasts (Fig. S5). *TNF* was expressed in macrophages, T cells and B cells. Sub-clustering analysis revealed that human RA Fibro_9 was likely to be comprised of tissue-destructive synovial fibroblasts based on the expression of *TNFSF11* and *MMP13* (Fig. 5C).

The calculation of module scores of mouse CIA fibroblasts markers by AddModuleScore confirmed that human RA Fibro_9 was homologous to tissue-destructive mouse CIA fibro_3 (Fig. 5D). RA Fibro_2, 7 and 8 may be inflammatory synovial fibroblasts based on the expression of *IL6* and *CCL2* (Fig. 5C and D)*.* Among the RA synovial myeloid cell subsets, Myel_2 was comprised of *ITGAM*^+^*CD163*^+^*MERTK*^hi^ tissue-resident macrophages homologous to mouse CIA myel_c1-4 (Fig. 5E, S6).

We further examined the expression of JAK/STAT pathway cytokine receptors in each cell type in the RA synovium. Human OSM signals via both OSMR-gp130 and LIFR-gp130 heterodimers. *OSMR, IL6ST* and *LIFR* were primarily expressed in RA Fibro_2, 7, 8, 9, while *IL6R* was mainly expressed in RA Myel_2 (Fig. 5F). Cell–cell communication analysis confirmed the interaction between RA Fibro_8, 9 and RA Myel_2 (Fig. 5G)*.* In particular, OSM [RA Myel_2]—OSMR [RA Fibro_2, 7, 8, 9], OSM [RA Myel_2]—LIFR [RA Fibro_2, 7, 8, 9] as well as IL-6 [RA Fibro_2, 7, 8]—IL-6R [RA Myel_2] and RANKL [Fibro_9]*—*RANK [RA Myel_2] interactions underlie the macrophage-synovial fibroblast circuit (Fig. 4H). Taken together, these results suggest that, similar to CIA in mice, the OSM-IL-6 circuit may underlie the pathogenic macrophage-synovial fibroblasts interplay in RA.

### OSM signaling in synovial fibroblast promotes inflammation and joint destruction by activation of inflammatory and tissue-destructive synovial fibroblasts

**Fig. 6:**
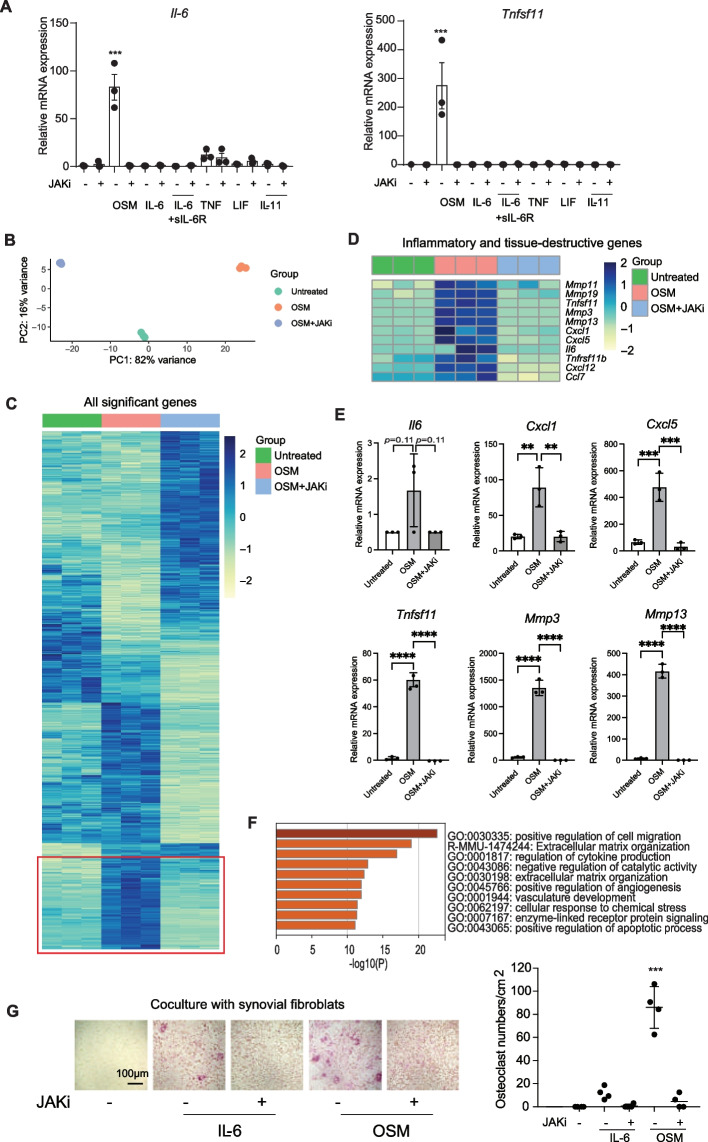
The effect of OSM and the JAK inhibitor on inflammatory and tissue-destructive properties of synovial fibroblasts. **A** *Il6* and *Tnfsf11* (RANKL) mRNA expression in mouse CIA synovial fibroblasts stimulated with indicated cytokines in the presence and absence of the JAK inhibitor (*n* = 3 per each condition). **B** Principal Components Analysis (PCA) plot of bulk mRNA sequencing of synovial fibroblasts in the three groups: untreated, OSM, OSM + JAKi treatments. **C** Heatmap of all significant gene expression. The genes which are significantly upregulated by OSM and then downregulated by the addition of the JAK inhibitor are marked in red. **D** Heatmap of selected inflammatory and tissue-destructive gene expression across the 3 groups. **E** mRNA expression of *Il6, Cxcl1, Cxcl5, Tnfsf11, Mmp3 and Mmp13* across the 3 groups by qPCR analysis. **F** Gene Ontology (GO) Term Enrichment analysis using genes that were significant upregulated in OSM and downregulated by the addition of the JAK inhibitor (JAKi). Top 10 GO Terms are shown. **G** The JAK inhibitor inhibited osteoclastogenesis induced by CIA synovial fibroblasts under OSM stimulation. All data are expressed as the mean ± S.D. **p < 0.01, ***p < 0.001, ****p < 0.0001 by 1-way ANOVA with the Holm-Sidak multiple-comparison test (A, E and G), significant vs. all other groups (**A** and **G**)

We next examined the expression of genes affected by OSM in synovial fibroblasts. Among the gp130 family cytokines, OSM most potently induced RANKL and IL-6 expression by CIA synovial fibroblasts (Fig. 6A). We confirmed that OSM-mediated phosphorylation of JAK1 was inhibited by the JAK inhibitor in synovial fibroblasts (Fig. S7). The JAK inhibitor completely inhibited this OSM-induced RANKL and IL-6 expression (Fig. 6A). In order to analyze comprehensively, we performed RNA-seq analysis on synovial fibroblasts treated with OSM or OSM + the JAK inhibitor (Fig. 6B and C). We found that inflammatory genes such as *Il6*, *Cxcl1* and *Cxcl5*, as well as tissue-destructive genes such as *Tnfsf11*, *Mmp3* and *Mmp13* were upregulated by OSM stimulation and this was abrogated by the addition of the JAK inhibitor (Fig. 6D and E). GO analysis indicated the genes related to cell migration, extracellular matrix organization and cytokine production were upregulated by OSM and downregulated by the JAK inhibitor (Fig. 6F). These results suggest that JAK inhibitors inhibit OSM-driven activation of inflammatory and tissue-destructive synovial fibroblasts.

It was previously reported that although JAK inhibitors do not exert direct effects on osteoclast precursors, they do inhibit osteoclastogenesis when osteoclast precursors are co-cultured with osteoblasts by inhibiting osteoblast RANKL expression [14, 15]. However, the effect of JAK inhibitors on synovial fibroblast-mediated osteoclastogenesis has not been well investigated. Here is shown that synovial fibroblasts stimulated with OSM induced osteoclastogenesis and that the JAK inhibitor abrogated this osteoclastogenesis by inhibiting synovial fibroblast-RANKL expression (Fig. 6G, Fig S8).

### The critical role of fibroblast-OSM signaling in arthritis as a key target of JAK inhibitors in vivo

**Fig. 7:**
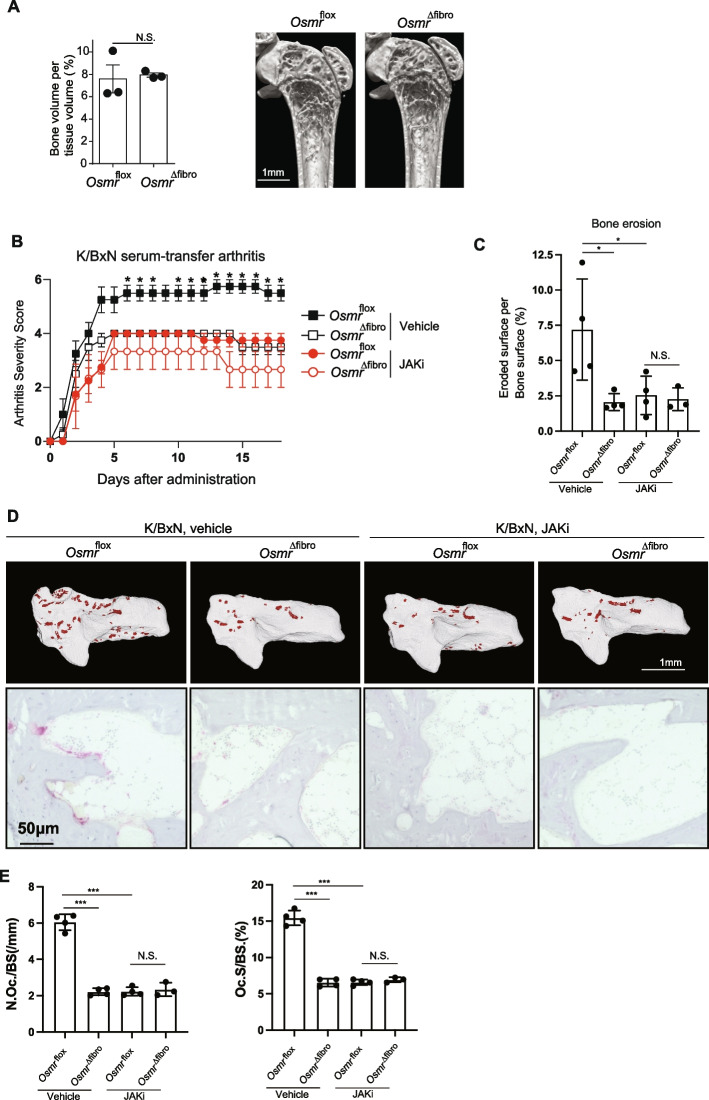
The critical role of fibroblast-OSM signaling in arthritis as a key target of JAK inhibitors in vivo*.* **A** Bone volume per tissue volume (left) and representative micro-CT images of the femur of untreated Osmr^flox^ and Osmr^∆fibro^ mice (*n* = 3, respectively, right). Scale bar: 1 mm. **B** Arthritis score of the hind paws of K/BxN serum-transfer arthritis Osmr^flox^ (vehicle, *n* = 4), Osmr^∆fibro^ (vehicle, *n* = 3), Osmr^flox^ (the JAK inhibitor, *n* = 4), Osmr^∆fibro^ (the JAK inhibitor, *n* = 3). **C** Eroded surface per bone surface of the calcaneus of Osmr^flox^ (vehicle, *n* = 4), Osmr^∆fibro^ (vehicle, *n* = 3), Osmr^flox^ (the JAK inhibitor, *n* = 4), Osmr^∆fibro^ (the JAK inhibitor, *n* = 3) in K/BxN serum-transfer arthritis. **D** Representative μCT images (upper panel) and TRAP staining (lower panel) of the calcaneus of K/BxN serum-transfer arthritis mice. The red colored area indicates cavities (upper) and TRAP^+^ osteoclast (lower). Scale bar: 1 mm (upper) and 50 µm (lower). **E** The number and surface of TRAP^+^ multinucleated cells per bone surface in the calcanueous of Osmr^flox^ (vehicle, *n* = 4), Osmr^∆fibro^ (vehicle, *n* = 3), Osmr^flox^ (the JAK inhibitor, *n* = 4), Osmr^∆fibro^ (the JAK inhibitor, *n* = 3) in K/BxN serum-transfer arthritis. All data are expressed as the mean ± S.E.M. *p < 0.05, **p < 0.01, ***p < 0.001 by unpaired Student’s t test (**A**), 2-way ANOVA with the Tukey’s multiple-comparison test (B, C, E), N.S., not significant

To elucidate the role of synovial fibroblast OSM signaling in arthritis, we generated *Col6a1*-Cre *Osmr*-floxed mice (hereafter *Osmr*^∆Fibro^ mice) in which OSMR is specifically deleted in synovial fibroblasts in joints. The femoral bone volume of femur was comparable between *Osmr*^∆Fibro^ and the control *Osmr*^flox^ mice, indicating that *Osmr*^∆Fibro^ mice undergo normal bone remodeling under physiological conditions (Fig. 7A).

To elucidate the primary role of synovial fibroblast OSM signaling as a drug target in vivo*,* we induced K/BxN serum-transfer arthritis in *Osmr*^∆Fibro^ mice and administered the JAK inhibitor. Both joint swelling and bone erosion were impaired in *Osmr*^∆Fibro^ mice compared to the control *Osmr*^flox^ mice in K/BxN serum-transfer arthritis, indicating that OSM signaling in synovial fibroblasts is important for both inflammation and bone destruction in arthritis (Fig. 7B-D). Administration of the JAK inhibitor ameliorated both joint swelling and bone erosion in the control *Osmr*^flox^ mice, while the JAK inhibitor had no effect on *Osmr*^∆Fibro^ mice under arthritic conditions (Fig. 7B-D). The number of osteoclasts was decreased in *Osmr*^∆Fibro^ mice compared to the control *Osmr*^flox^ mice in arthritis. In addition, while administration of the JAK inhibitor impaired osteoclastogenesis in the control mice, it had no effect on *Osmr*^∆Fibro^ mice (Fig. 7D, E). These results indicated that synovial fibroblast-OSM signaling plays a key role in arthritis as a drug target of JAK inhibitor in vivo.

Taken together, OSM signaling in synovial fibroblast plays an important role in inflammation and joint destruction in arthritis in vivo by activation of inflammatory and tissue-destructive synovial fibroblasts. The combination of scRNA-seq analysis of mice and humans with genetic loss experiments clarified that the OSM-driven synovial macrophage-fibroblast interaction is indispensable for both the pathogenesis and the crucial drug target in arthritis.

## Discussion

The triangular immune cell-synovial fibroblast-bone cell interaction underlies RA pathogenesis. Recent scRNA-seq analyses have revealed the active pathogenic cell subsets in various diseases, including RA. In particular, the pathological significance and the polarization mechanism of distinct synovial fibroblast subsets have attracted considerable attention. However, the cell–cell interactions which critically are important for RA pathogenesis have not been fully determined. Targeted synthetic DMARDs (tsDMARDs) such as JAK inhibitors have been introduced into the clinic and are widely used for multiple diseases, including RA. However, in vivo target cells and/or the signaling pathways of such tsDMARDs remain unidentified, although in vitro experiments have shown that JAK inhibitors inhibit activation of various cells including T cells, B cells, dendritic cells and synovial fibroblasts [6, 12, 13, 39–42].

The expression of OSM is reportedly upregulated in autoimmune diseases, including RA [42, 43, 44]. Although it was reported that overexpression of OSM exacerbates cellular infiltration in synovium, the major cellular source of OSM or OSMR was unclear and the pathogenic relevance of OSM signaling in vivo has not been elucidated [26, 27, 45]. In vitro experiments showed JAK inhibitors impaired the expression of particular cytokines such as IL-6 and MCP-1 in synovial fibroblasts stimulated with OSM, but the importance of OSM signaling in synovial fibroblasts as the major target of JAK inhibitor in vivo has never been identified. In addition, a comprehensive analysis of genes whose expression was impaired by JAK inhibitors in OSM-stimulated synovial fibroblasts has not been reported [39–42].

Fibroblasts, originally recognized as structural cells, play an important role in tissue homeostasis and various diseases, including RA, cancer and colitis [46]. These findings have attracted considerable attention and given rise to a new discipline, stromal immunology. However, there are as yet no approved therapies which were generated in order to target directly synovial fibroblasts. scRNA-seq analysis revealed the existence of distinct fibroblast populations, inflammatory fibroblasts and tissue-destructive fibroblasts. Since synovial fibroblasts play a key role in RA pathogenesis, synovial fibroblasts have been considered a promising therapeutic target in RA [4, 21, 46]. Inflammatory synovial fibroblasts reside in the sublining layer, whereas tissue-destructive synovial fibroblasts reside in the lining layer of the synovial membrane. Recent studies have shown NOTCH signaling is an important driving factor of inflammatory synovial fibroblasts [20]. We reported that ETS1 is critical for the polarization of tissue-destructive synovial fibroblasts [21]. Since endothelial cells provide NOTCH signaling and the hypoxic conditions in the lining layer of the arthritic synovium may induce ETS1 expression, it is suggested that spatial regulation may be involved in the polarization of distinct synovial fibroblast subsets. However, little is known about the shared signaling pathways which lead to the polarization of inflammatory and tissue-destructive synovial fibroblasts [46, 47].

Based on computational analysis together with biological studies, we found that OSM is the common activator of both inflammatory and tissue-destructive synovial fibroblasts. These activated synovial fibroblasts exacerbate inflammation and bone destruction in arthritis by interacting with macrophages. We clarified that OSM from macrophages promotes the pathogenic macrophage-synovial fibroblast circuits in arthritis in both mice and humans and that JAK inhibitors mainly target the fibroblast-OSM signaling, which is critical for arthritic inflammation and bone destruction in vivo. Thus, therapeutic manipulation of the OSM signaling pathway could be a promising approach to the treatment of RA. There is a paper reporting a failure of a clinical trial for an anti-OSM antibody in RA possibly due to the low binding affinity of the antibody to OSM [48]. It is necessary to evaluate the efficacy of suppression of OSM in RA using better antibodies or other methods in the future [45]. Since anti-OSM antibodies ameliorated murine arthritis when administered before or immediately after the onset of arthritis [49], it is possible that OSM signaling may be important at the earlier phase of autoimmune arthritis rather than the later phase. It will be intriguing to examine whether anti-OSM antibodies are effective in early-stage patients. OSM blockage can be a promising therapeutic strategy if we select appropriate time points of drug administration and appropriate combinations with other therapies. Considering that both JAK inhibitors and IL-6 blockade are effective in RA, it is plausible that JAK inhibitors mainly target IL-6 signaling and the inhibitory effect of JAK inhibitor can be explained by the inhibition of the IL-6 signaling. However, it is reported that a JAK inhibitor induced greater improvements compared with IL-6 blockade in bDMARD-naïve patients active RA refractory to MTX [50]. In addition, it is reported that JAK inhibitors were effective for RA patients who were refractory to multiple b/tsDMARDs including IL-6 blockade [51]. These reports suggest that IL-6 may be not the sole target of JAK inhibitors and imply the importance of other signaling pathways such as OSM signaling.

It is important to clarify positional relationships among OSM^+^ macrophages, OSMR^+^ fibroblasts, IL-6^+^ fibroblasts and IL-6R^+^ macrophages in the inflamed joints. We confirmed expression of OSM and OSMR in arthritic synovium, however, it was technically difficult to detect all the markers simultaneously by immunohistochemistry (Fig. S9). Alternatively, we reanalyzed special transcriptomic data [52]. Although only a few OSM^+^ cells were detected, it is suggested that OSM^+^ macrophages and OSMR^+^ fibroblasts may interact each other in the lining layer (Fig. S9). Technical advances in special transcriptomics will help us understand the spatiotemporal relationships among OSM^+^ macrophages, OSMR^+^ fibroblasts, IL-6^+^ fibroblasts and IL-6R^+^ macrophages in the inflamed synovium during the course of arthritis in future.

Considering that the OSMR signaling is important for the polarization of both inflammatory and tissue-destructive synovial fibroblasts, it may be desirable that JAK inhibitors could be administered at the early stage of RA to inhibit the polarization of pathogenic synovial fibroblasts. We have combined scRNA-seq analysis of drug-treated tissues and analysis of mice with cell-specific gene deletion, which strategy will further elucidate the key cell–cell interactions in various diseases.

## Conclusion

We often use drugs even if we do not know exactly how they work. Clarification of their mode of action in the body will contribute to development of therapeutic strategies and understanding disease pathogenesis. JAKs are ubiquitously expressed in various types of cells and regulate cellular functions. JAK inhibitors are effective in rheumatoid arthritis, but the in vivo targets remained unclear. Here, by scRNA-seq analysis of drug-treated tissues and analysis of genetically modified mice with JAK inhibitor administration, we clarified that OSM signaling in fibroblasts is one of the main targets of JAK inhibitors and plays an important role in arthritis. OSM, produced by macrophages, activates fibroblasts which exacerbate inflammation and bone destruction. Thus, the OSM-driven macrophage-fibroblast interaction is proven to govern arthritis pathogenesis.

## Material and methods

### Study design

This study was designed to identify the synovial cell interactions and signaling pathways in autoimmune arthritis by analyzing mice and human scRNA-seq datasets. We induced CIA on DBA/1J mice followed by administration of a JAK inhibitor, upadacitinib. Mouse synovial cells were prepared from the synovium of untreated, CIA or CIA + the JAK inhibitor mice for scRNA-seq analysis. The computational analyses included scRNA-seq, pseudo bulk RNA-seq, trajectory, RA scoring, cytokine target genes, cell–cell interaction and enrichment analysis. Published human RA synovial scRNA-seq datasets were integrated and analyzed for a comparison to the mouse data. RNA-seq analysis of CIA synovial fibroblasts was performed to evaluate the effect of OSM on synovial fibroblasts and the JAK inhibitor on synovial fibroblasts stimulated with OSM. In vitro osteoclastogenesis was performed to confirm the destructive activity of synovial fibroblasts stimulated with OSM and the effect of the JAK inhibitor on these cells. *Col6a1*-Cre *Osmr*-floxed and the control *Osmr*-floxed mice were induced of K/BxN serum-transfer arthritis to evaluate the role of synovial fibroblast-OSMR in arthritis in vivo. The study was approved by the Institutional Review Board at The University of Tokyo. We chose the sample size based on our prior experience and the standards in the relevant fields published in studies [18, 19] and remain in compliance with ethical guidelines to minimize the experimental animals.

### RA synovium

Human synovial tissue specimens were obtained from RA subjects undergoing joint replacement surgery or synovectomy at the Tokyo University Hospital. All the subjects with RA fulfilled the 2010 American College of Rheumatology European League Against Rheumatism criteria for the classification of RA and provided written informed consent. This study was approved by the Institutional Review Board at The University of Tokyo.

### Mice

All animals were maintained under specific pathogen-free conditions. The experiments were approved by the Institutional Review Board at The University of Tokyo. *Col6a1*-Cre mice were previously described [53]. B6;129-Osmrtm1.1Nat/J (*Osmr*^flox^/^flox^) mice were purchased from The Jackson Laboratory (Bar Harbor, Maine, USA). Fibroblast-specific *Osmr*-deficient (*Osmr*^∆Fibro^) mice were generated by breeding *Osmr-*floxed mice with *Col6a1*-Cre mice.

### CIA and K/BxN serum-transfer arthritis

For CIA, 6- to 8-week-old DBA/1J sex-matched mice (Charles River Laboratories Japan) were used. An emulsion which consisted of 50 μl of chicken type II collagen (Sigma-Aldrich, 4 mg/ml) and 50 μl of adjuvant into the base of the tail at two sites. We added heat-killed Mycobacterium tuberculosis H37Ra (Difco Laboratories, 4 mg/ml) in incomplete Freund’s adjuvant (IFA) (Difco Laboratories). Three weeks after the primary immunization, mice were challenged with the same emulsion as the primary immunization. We judged the development of arthritis in the joint using the following criteria: 0, no joint swelling; 1, swelling of one paw joint; 2, mild swelling of the wrist or ankle; 3, severe swelling of the wrist or ankle. The scores for all of the joints of forepaws and hind paws, wrists and ankles were totaled for each mouse (with a maximum possible score of 12 for each mouse).

For K/BxN serum-transfer arthritis, mice were administered 150 μl pooled serum collected from arthritic K/BxN mice (8 weeks) by intraperitoneal injection on day 0, day 2 and day 9 (analyzed on day 20). The hind paws of mice were monitored every day for signs of arthritis. The animal numbers used in each experiment are described in the corresponding figure legends.

### Administration of a JAK inhibitor

CIA mice were administered a JAK inhibitor, upadacitinib (Selleck, 12 mg/kg) in 0.5% methylcellulose, 0.025% Tween 20 solution [17] or vehicle by oral gavage twice a day from d7 to d21 after the 2nd immunization. K/BxN serum-transfer arthritis mice were administered the JAK inhibitor, upadacitinib (Selleck, 24 mg/kg) in 0.5% methylcellulose, 0.025% Tween 20 solution or vehicle by oral gavage twice a day from d0 to d20.

### Preparation of synovial fibroblasts

The synovial tissue was obtained from the knee joints of CIA mice 3w after 2nd immunization. The synovium was minced and incubated with 5 mg/ml collagenase type II (CLS-2, Worthington Biochemical corporation) in serum-free DMEM (Life Technologies) for one hour at 37 ℃, filtered, washed, and cultured in DMEM (Life Technologies) supplemented with 10% FBS. Cultured fibroblasts during the fourth to seventh passages were used for the experiments.

### Immunoblot analysis

CIA synovial fibroblasts cultured under serum-free conditions were pretreated with upadacitinib (Selleck, 500 nM) for 2 h and stimulated with OSM (R&D, 495-MO, 20 ng/ml) for 20 min. Cells were washed by PBS and lysed with a lysis buffer (RIPA buffer, 16,488–34, Nacalai tesque) with gentle agitation for 20 min at 4℃.　Cell lysates was subjected to 5–12% SDS polyacrylamide gel electrophoresis, and then transferred to a PVDF membrane. After blocking for one hour at room temperature, membranes were incubated phospho-JAK1 antibody (3331, Cell Signaling Technology), anti-JAK1 antibody (3344, Cell Signaling Technology) or anti-β-actin antibody (Cat 3700, Cell Signaling Technology) overnight at 4 ℃. Membranes were subsequently incubated for 1 h with anti-rabbit IgG, HRP-linked antibody (7074S, Cell Signaling Technology) and anti-mouse IgG, HRP linked antibody (7076S, Cell Signaling Technology) respectively. Immunoblot analysis was performed with ImmunoStar blotting kit (292–69,903, Wako).

### Immunohistochemistry

Sections were deparaffinized and rehydrated followed by antigen retrieval in HistoVT One solution (06380–05, Nacalai tesque) at 93 ℃ for 15 min. Sections were washed in Tris buffered saline (TBS) and endogenous peroxidase activity was blocked by incubating the sections in 3% H_2_O_2_ (diluted in TBS) for 20 min. Sections were incubated with blocking one histo solution (06349–64, Nacalai tesque) for 60 min at room temperature and then incubated with the primary antibody overnight at 4℃. Sections were subsequently incubated for 1 h with HRP-conjugated Affinipure anti-goat IgG(H + L) (SA00001-4, Proteintech) at 4℃, followed by detection with DAB Chromogen/Substrate kit (ACT500-IFU, ScyTek Laboratories). Samples were counterstained with Gill's haematoxylin (Cod. 30,002, Muto Pure Chemicals Co. Ltd). Images of the stained tissues were captured using BZ-9000 (KEYENCE, JAPAN).

### Reanalysis of spatial transcriptomics

Spatial transcriptomics data [52] (https://www.immport.org/shared/study/SDY2213) was re-analyzed by Seurat R package (v5). RA3 section data was filtered with min cells/ gene > 3 and min genes/cell > 200. PCA was performed on the normalized expression of highly variable genes, with the top 30 PCs retained. SpatialFeaturePlot function was then used to visualize the expression of OSM, IL-6, OSMR, and IL-6R on the spatial transcriptomics slide.

### Single-cell RNA-seq analysis

Single-cell RNA-seq analysis was performed by the 10 × Genomics Chromium system. Synovial cells from knee joints were prepared from 9 healthy mice, 3 CIA mice, and 6 the JAK inhibitor treated mice and captured with the 10 × Genomics Chromium system (3,000 cells assigned). Sequencing libraries were generated using a 10 × Genomics Single-cell 3′ Solution (v.3) kit and then subjected to Illumina sequencing (HiSeq 4000). Alignment and quantitation of sample count matrices were performed using the 10 × Genomics Cell Ranger pipeline (v.3.0) and mouse reference sequences (version mm10) as indicated in the manufacturer’s protocol.

### Single-cell RNA-seq computational methods

Downstream analysis was performed using the Seurat R package (v.3) with a primary input of 3,000 cells/group, as in our previous studies [54, 55]. Genes were primarily filtered out when expressed in < 3 cells or from cells with < 200 genes. Cells with greater than 5% mitochondrial reads and 7,500 nFeature_RNA were also excluded. After quality control, per-cell counts were integrated, normalized and scaled using standard and SCT transform functions. The first 30 principal components were retained for UMAP projection and clustering analysis using a graph-based clustering approach and modularity optimization techniques. After primary analysis, minor cell populations were excluded (e.g. cycling, neural and blood cells) and the cells were re-analyzed as above. We also integrated human RA synovial single-cell RNA-seq datasets from the dbGaP Study, Accession phs001529.v1.p1 [38], and the www.immport.org Study, Accession SDY998 [19], using Seurat v3 and a standard CCA procedure.

The FeaturePlot was used with the minimum and maximum cutoff values set at quantile 10 and quantile 90 to ﻿visualize single cells on a UMAP plot according to their gene expression. Cluster marker findings identified positive and negative markers of a single cluster﻿ using a Wilcoxon Rank Sum test, with an ﻿adjusted *p*-value < 0.05 and log2FC > 0.1 by Bonferroni correction using all genes in the dataset. The average gene expression of each condition of the mouse data in each cell type was also calculated by the AverageExpression function followed by the identification of differentially expressed genes across conditions with log2FC > 0.25 (pseudo bulk RNA-seq analysis).

To compute the RA scores in mouse synovial cells at the single-cell level, we constructed a gene list based on the mmu05323 KEGG pathway (rheumatoid arthritis, mouse, Metacarp v3.5) and Seurat function AddModuleScore. Inflammatory and destructive scores were calculated using an inflammatory gene list (*Il6, Cxcl1, Cxcl2, Cxcl5, Cxcl10, Cxcl12, Ccl2, Ccl7*) and destructive gene list (*Tnfsf11, Mmp2, Mmp3, Mmp9, Mmp11, Mmp13, Mmp14, Mmp19*). These genes were found in the mmu05323 KEGG pathway gene list and highly expressed in fibroblast clusters. For each individual cell, the average expression of each of 85 genes was calculated. Genes were binned (nbin = 24) based on the averaged expression and 100 control genes were randomly selected from each bin. Trajectory analysis was performed using clusters of cells to uncover the global structure, and this structure was converted into smooth lineages represented by one-dimensional variables by Slingshot (v1.2) [56, 57]. The process included identifying the global lineage structure with a cluster-based minimum spanning tree and fitting simultaneous principal curves to describe each lineage. The single-cell data matrix, the dimensionality reduction produced by UMAP and the cluster identified by Seurat were used to establish the cell trajectories of mouse fibroblasts and myeloid cells.

We used the database of target genes to analyze the number of JAK/STAT cytokine target genes. For each cytokine, we examined the microarray and/or RNA-seq data and made a list of the significantly upregulated genes (Data file S3). These cytokine target gene lists were used to calculate the average expression levels (the signature score: the combined expression score of the genes in each target set) in each cluster at the single-cell level using Seurat's AddModuleScore function. In the mouse single-cell data, we used the top 50 (ranked by fold change) cytokine target genes of 15 gene sets, including IL-2, IL-4, IL-15, IL-21 [28] IL-7 [29], IL-6 [30], OSM [31], IFN-α/β/γ [32], IL-10 [33], IL-22 [34], GM-CSF [35], EPO [36] and GH [37]. Cells that had the highest score across all modules and those in the 95% quantile of the highest score were assigned to the related cytokine targets. Cells of lower score cells were categorized as "other cells". Similarly, AddModuleScore was also used to identify cell populations which are resemble to mouse fibroblasts and myeloid cells under CIA conditions, in RA data using the significant markers found in each CIA cell cluster.

### Pathway and cell communication analysis

Enrichment clustering and Multi-gene-list meta-analysis were performed based on terms across different ontology sources, such as GO and KEGG by using Metascape (v3.5) [58]. We then identified all of the statistically enriched terms, accumulative hypergeometric p-values and enrichment factors for each gene set, which were then used for filtering. Cell–cell interaction analysis was performed using curated receptors, ligands and their interactions from the single-cell transcriptomics data. We used the Python package CellPhoneDB (v.2.1.7) [24, 59, 60]. Single-cell RNA-seq mouse and human data were processed with cluster information and user-specific custom interactions (JAK/STAT signaling cytokines, TNF, IL17, TNFSF11, and their receptors).

The gene expression was also processed for GSEA (v.4.1) in 19 pseudo bulk RNA-seq data from 19 mouse clusters of single-cell RNA-seq data under CIA and the JAK inhibitor conditions [61, 62]. GSEA was run with 1000 permutations for each of the gene sets and selected with the FDR adjusted *P*-value < 0.05 for normalized enrichment score (NES) in the Diff_of_Classes mode (which used the difference of class means to calculate fold change for log scale data). We used 14 gene sets available in MSigDB (v7.5) and the KEGG GENES Database, including Hallmark interferon alpha response (M5911), Hallmark interferon gamma response (M5913), Hallmark IL2 STAT5 signaling (M5947), Hallmark IL6 JAK STAT3 signaling (M5897), WP IL1 signaling pathway (M39346), WP oncostatin M signaling pathway (M39562), WP IL4 signaling pathway (M39720), WP IL17 signaling pathway (M39560), WP TNF-alpha signaling pathway (M39662), KEGG JAK STAT signaling pathway (M17411), WP EPO receptor signaling (M39687), WP IL10 anti inflammatory signaling pathway (M39796), Reactome interleukin 7 signaling (M542).

### In vitro osteoclast differentiation

Osteoclast precursors were obtained by the culture of primary bone marrow cells purified from 7–10-week-old DBA/1J mice in α-MEM 10% FBS supplemented with 10 ng/ml M-CSF (R&D Systems) for 2 days. Osteoclast precursor cells (2 × 10^4^ cells/well) were then co-cultured with synovial fibroblasts (5 × 10^3^ cells/well) in the presence of 10 ng/ml M-CSF, 100 ng/ml OSM, 100 ng/ml IL-6, and the JAK inhibitor, upadacitinib (50–400 nM) for one week using a 96 well flat-bottom plate. The positive control was culture of osteoclast precursors with M-CSF and 50 ng/ml RANKL, and multinucleated cells (more than three nuclei) were counted by TRAP staining.

### Quantitative RT-PCR analysis

Real-time quantitative RT-PCR analysis was performed with a LightCycler (Roche) using SYBR Green (Toyobo). The level of mRNA expression was normalized by Gapdh expression. The following primers were used: *Gapdh*, 5 ´- TCCACCACCCTGTTGCTGTA-3 ´ and 5 ´-ACCACAGTCCATGCCATCAC-3 ´; *Tnfsf11*, 5 ´-AGCCATTTGCACACCTCAC-3 ´, and 5 ´-CGTGGTACCAAGAGGACAGAGT-3 ´; *Il6,* 5' CCGGAGAGGAGACTTCACAG 3', and 5' CAGAATTGCCATTGCACAAC 3'.

### Bone analysis

For microcomputed tomography analysis, the calcaneus of the arthritic mice was subjected to three-dimensional micro-computed tomography. CT scanning was performed using a ScanXmate-A100S Scanner (Comscantechno). Three-dimensional microstructural image data were reconstructed, and structural indices were calculated using TRI/3D-BON software (RATOC). For bone morphometric analysis, the calcaneus was fixed in 70% EtOH for 1 week. TRAP staining was performed to identify osteoclasts.

### Statistical analysis

Data were analyzed on GraphPad Prism software version 6.0 g and R studio software v4.1.2. The statistical tests, n values are *p* values all indicated in the figures and/or legends. Data are expressed as the mean ± S.E.M or S.D. P values were calculated using unpaired Student’s t-test, one-way ANOVA or two-way ANOVA with Holm-Sidak’s multiple or comparisons test (*p < 0.05; ** *p* < 0.01; ***p < 0.001; N.S., not significant, throughout the paper.

### Supplementary Information

Supplementary Material 1. 

## Data Availability

The scRNA-seq and bulk RNA-seq data used in this study are available in the public Gene Expression Omnibus (GEO) database under accession codes GSE192504 [21] and GSE224221. Codes are available at: https://github.com/namhuynhnc/OSM-paper

## Abbreviations

RA: Rheumatoid arthritis
CIA: Collagen-induced arthritis
μCT: Micro-computed tomography
JAK: Janus kinase
STAT: Signal transducer and activator of transcription
RANKL: Receptor activator of NF-κB ligand
bDMARD: Biologic disease modifying anti rheumatic drugs
tsDMARD: Targeted synthetic disease modifying anti rheumatic drugs
TNF: Tumor necrosis factor
IL: Interleukin
TRAP: Tartrate-resistant acid phosphatase
