# Supplementary material for: Oncostatin M-driven macrophage-fibroblast circuits as a drug target in autoimmune arthritis
Source: Inflamm Regen. 2024 Jul 31;44:36. doi: 10.1186/s41232-024-00347-0 (PMC11289929; doi:10.1186/s41232-024-00347-0)
Supplement: Supplementary file 1 — Supplementary Material 1. [file 41232_2024_347_MOESM1_ESM.pdf]

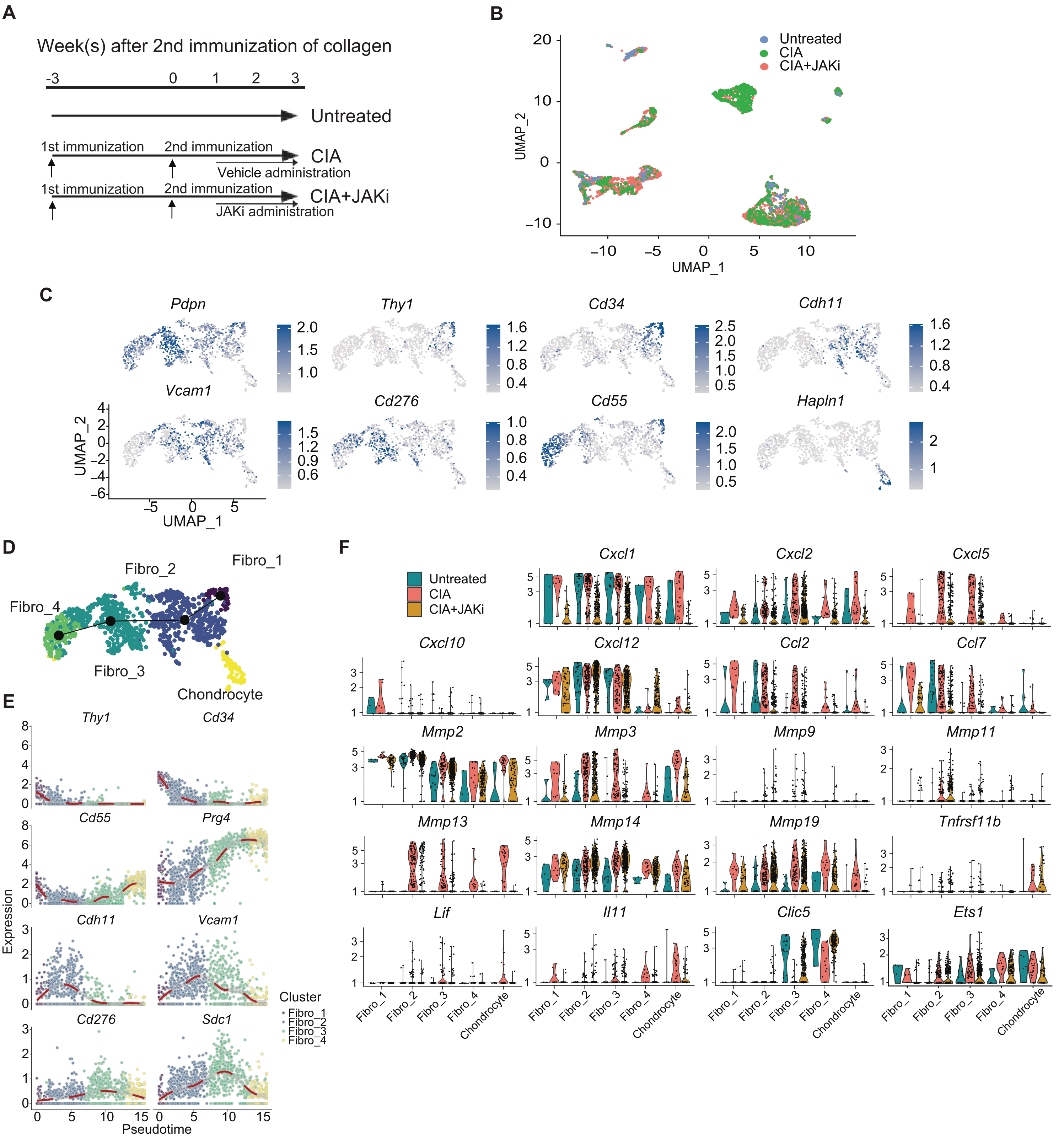

G

RA genes	*Angpt1, Atp6v1e1, Ctsk, Fos, H2-Aa, Il11, Il1b, Il6, Jun, Mmp3, Ccl2, Ccl20, Ccl3, Cxcl5, Tnf Vegfa, Atp6v1g1, Atp6v0b, Acta2, Adra2a, Bmp2, Ccr1, Dag1, F2r, Ccn2, Fn1, Fzd5, Lpar1, Igf1, Il1rn, Lif, Myc, Pak1, Psap, Sirpa, Ccl7, Sphk1, Thbs1, Zfp36, Synj2bp, Zeb2, Clcf1, Dact1, Nek6, Nenf, Dusp6, Rnf149, Sash1, Lamtor2, Cspg4, Dusp5, Cxcl1, Il1r1, Inhba, Cxc Cxcl14, Aplp2, Socs1, Ereg, Fbn1, Nedd9, Ddr2, Pim1, Tnfaip3, Tcim, Adarb1, Cblb, Axl, Irs Chchd2, Fzd1, Gch1, Gpx1, Grn, Hif1a, Ier5, Nr4a3, Ptgs2, Slc7a11, Herpud1, Rtn4, Glrx*
Inflammatory genes	*Il6, Cxcl1, Cxcl2, Cxcl5, Cxcl10, Cxcl12, Ccl2, Ccl7*
Destructive genes	*Tnfsf11, Mmp2, Mmp3, Mmp9, Mmp11, Mmp13, Mmp14, Mmp19*

Fig. S1. Characterization of mouse CIA synovial cells by scRNA-seq analysis.
(A) Experimental procedure to obtain the synovial tissues under the untreated, CIA and CIA+JAKi conditions for scRNA seq analysis. (B) Distribution of synovial cells under the untreated, CIA, and CIA+JAKi conditions. (C) Feature plots of the main markers of mouse synovial fibroblast sub-clusters. (D) Trajectory analysis of synovial fibroblast from fibro_1 via fibro_2, 3 to fibro_4. (E) Expression of the main markers during the trajectory in synovial fibroblasts. (F) Violin plots of inflammatory, destructive and RA-related genes in synovial fibroblast clusters under the untreated, CIA, and CIA+JAKi conditions. (G) List of RA, inflammatory and destructive genes.

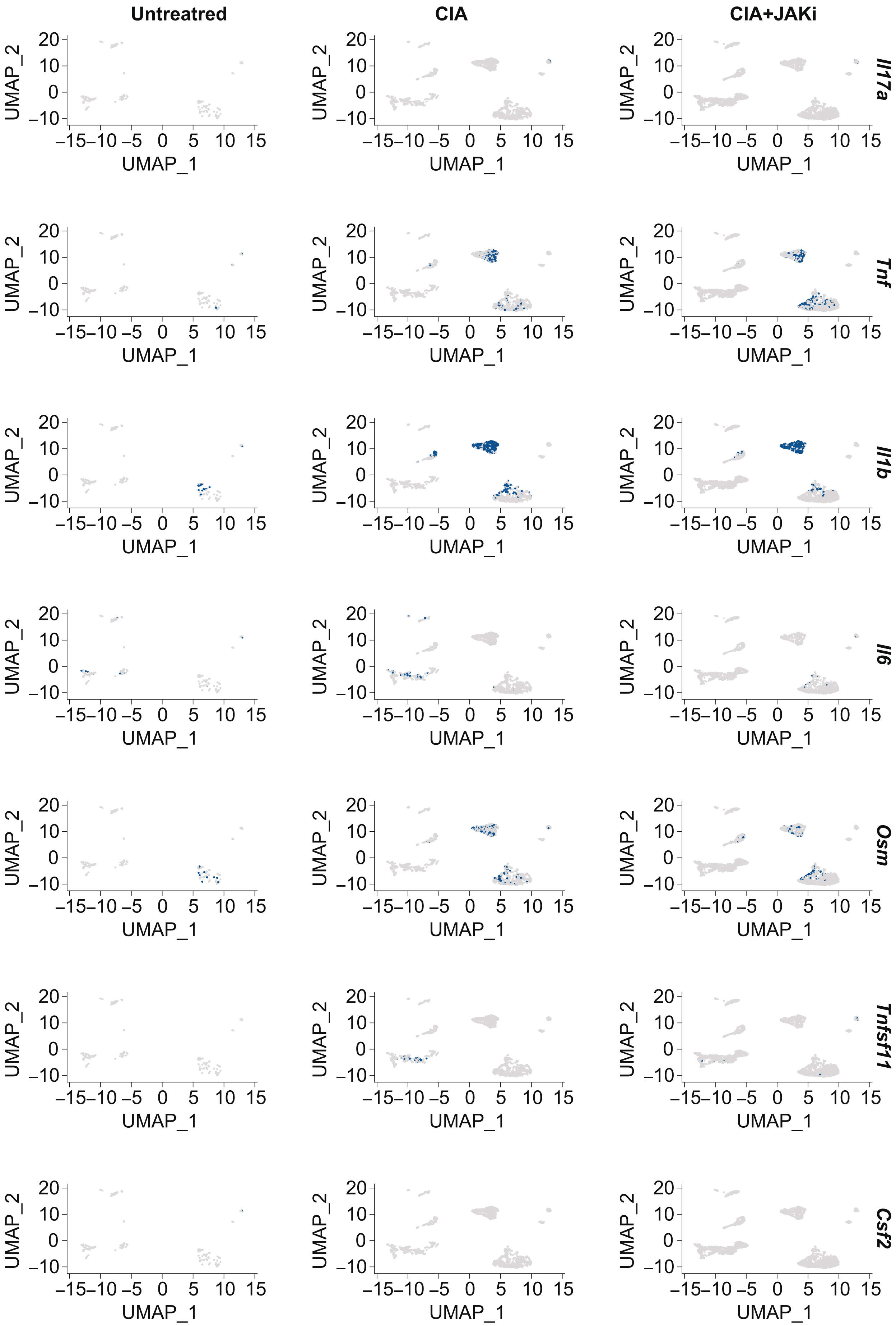

Fig. S2. Expression of the representative inflammatory and destructive genes in mouse synovial cells
Feature plot of the expression of *Tnf, Il1b, Il6, Osm, Tnfsf11* and *Csf2* in mouse synovial cells under the untreated (left), CIA (middle) and CIA+JAKi (right) conditions are shown.

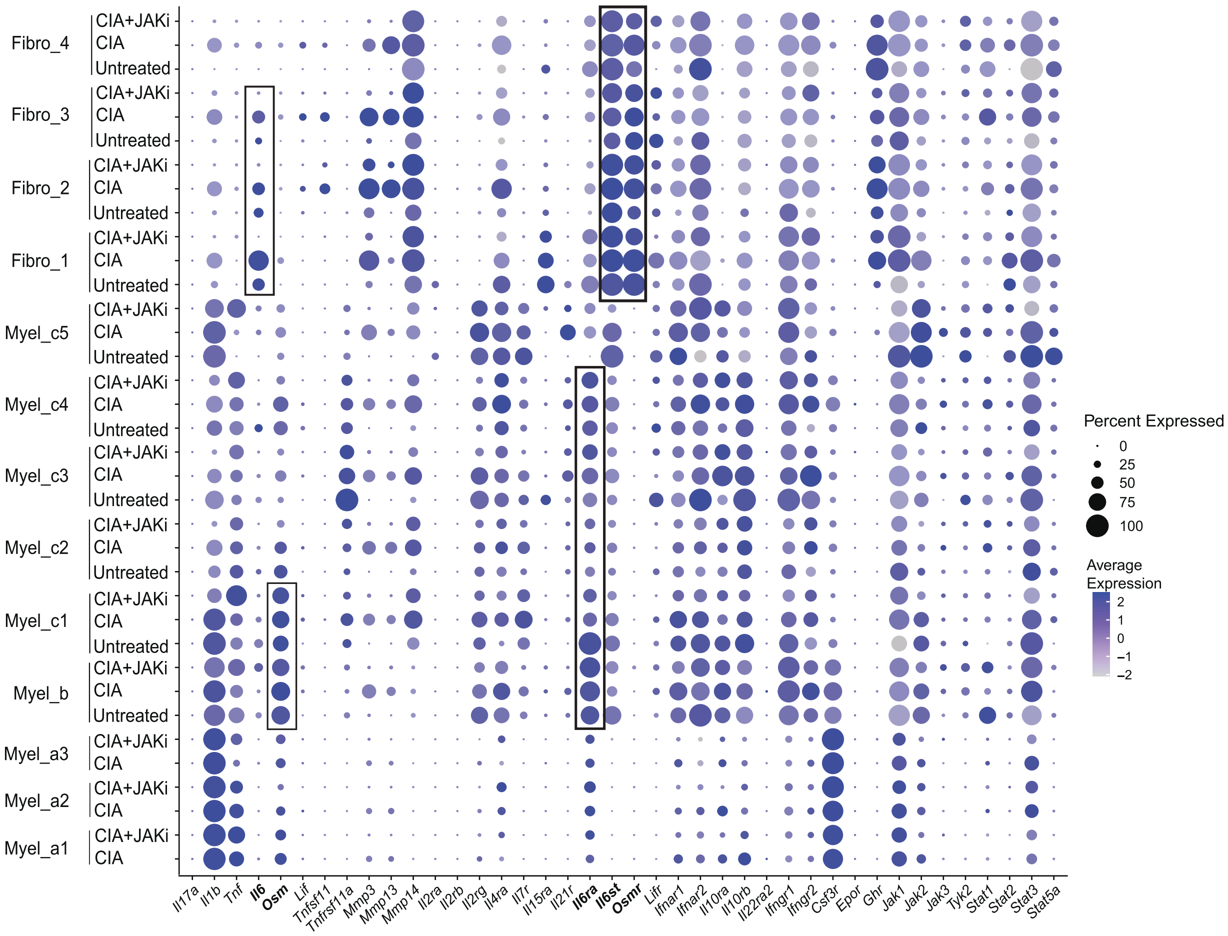

Fig. S3. Dot plot of average gene expression in CIA synovial cells.
Average expression of JAK/STAT cytokines and receptors as well as the representative inflammatory and desrtuctive genes in myeloid and synovial fibroblast sub-clusters under untreated, CIA, and CIA+JAKi conditions.

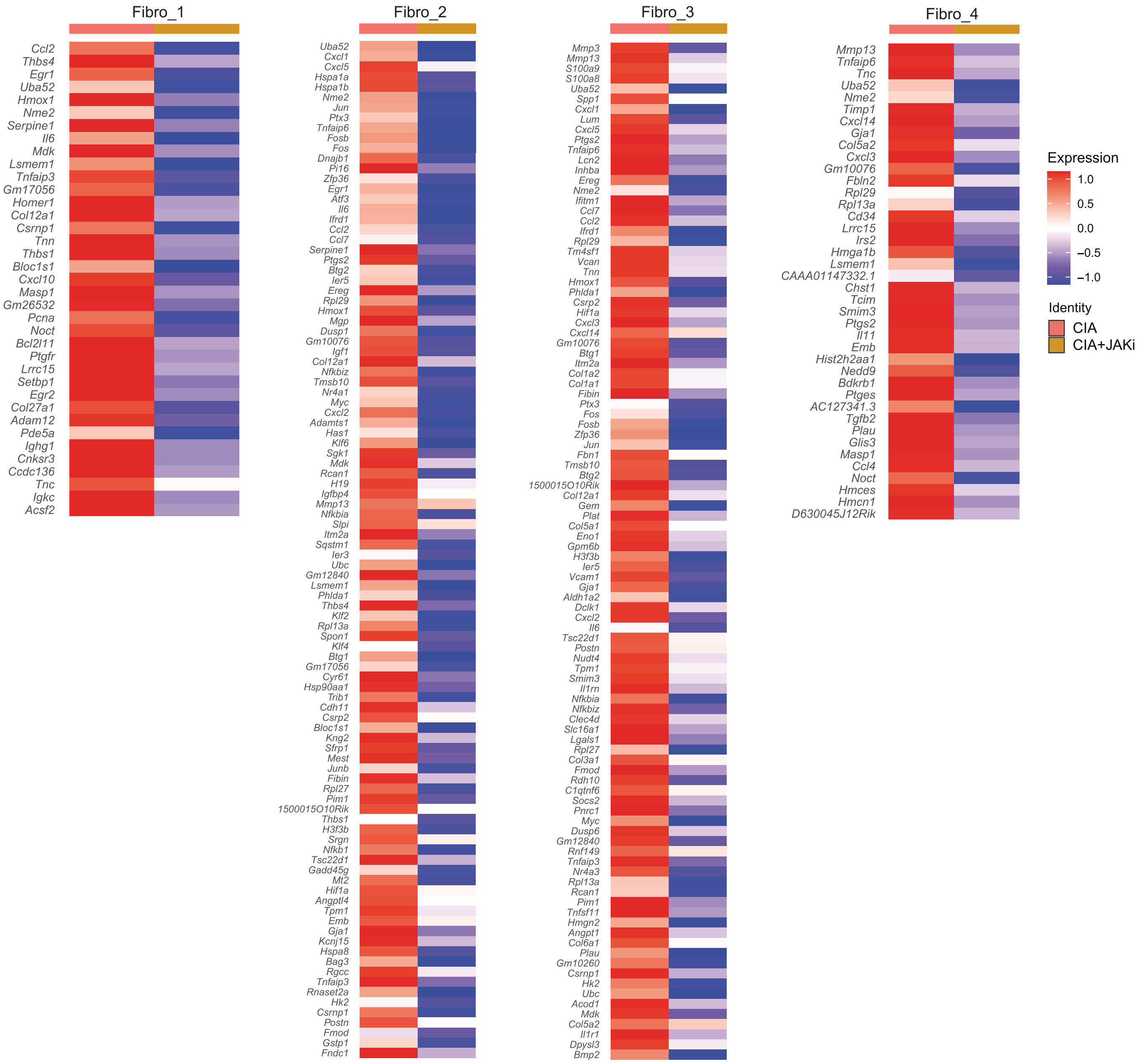

Fig. S4. Differentially expressed genes upregulated under CIA and downregulated under CIA+JAKi condtions in synovial fibroblst subpopulations

Differentially expressed genes (ranked by log2 fold change) upregulated under CIA (vs untreated) conditions and downregulated under CIA+JAKi (vs CIA) in synovial fibroblast subsets are shown.

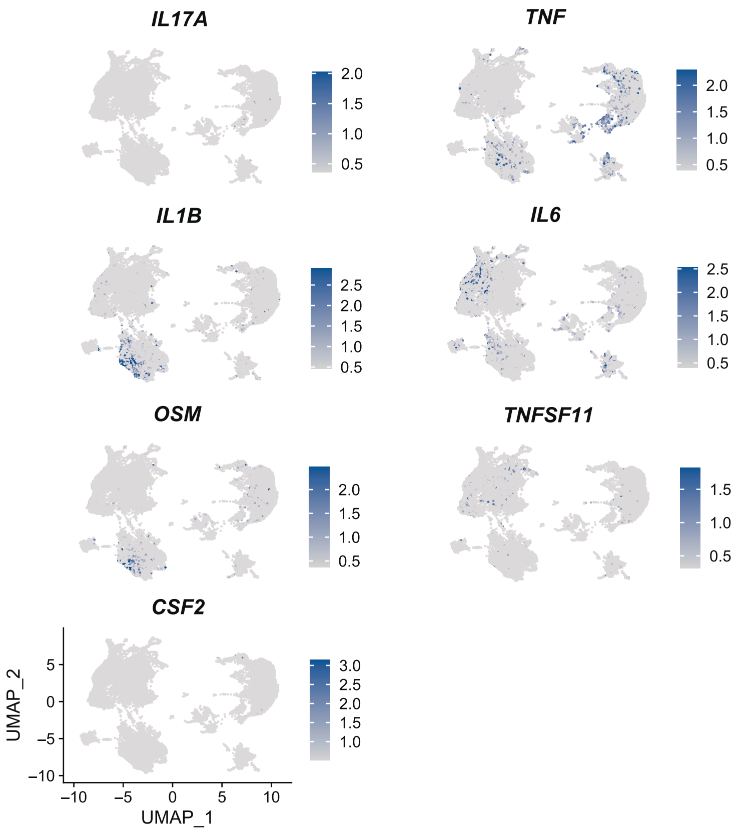

Fig. S5. Featureplots of expression of inflammatory and destructive genes in RA synovial cells.
Previously reported scRNA-seq data sets on RA synovial tissues (ref21,41) were integrated and used for analysis.

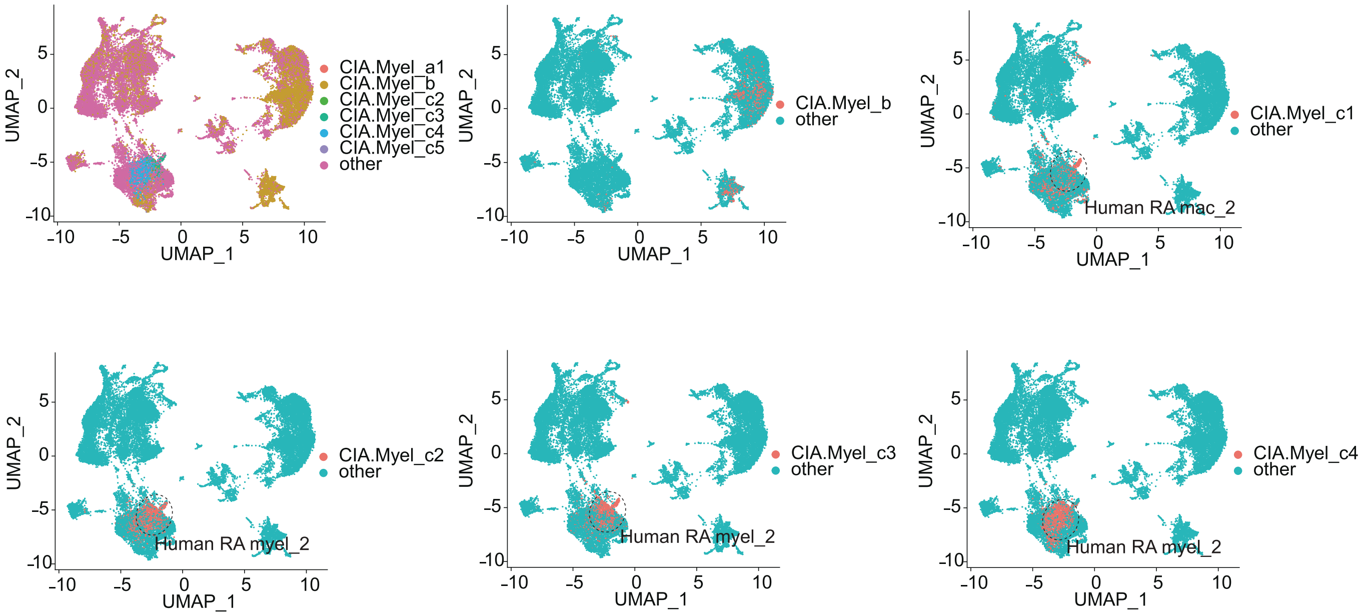

Fig. S6. Resemblance between CIA mouse myel_ c1-4 cells and RA myel_2.
Module scores of mouse CIA myeloid markers in human RA synovial cells were calculated by AddModuleScore.

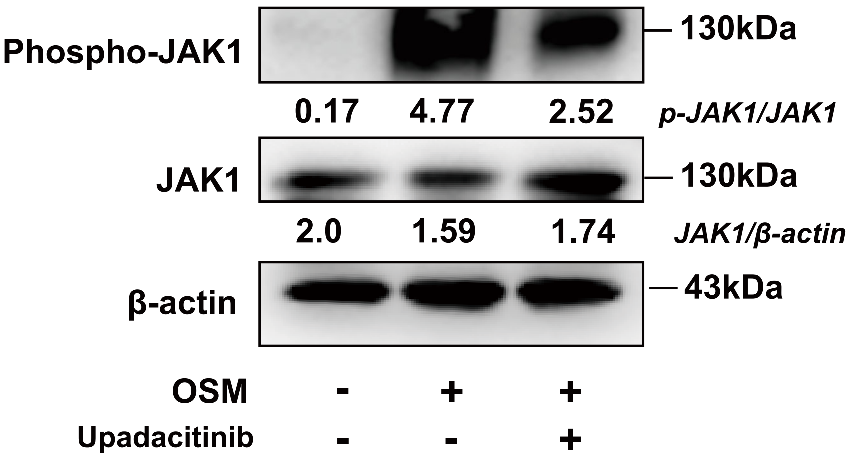

Fig. S7. Upadacitinib suppresses OSM-induced JAK1 phosphorylation in CIA synovial fibroblasts
Immunoblot analysis of CIA synovial fibroblasts pretreated with upadacitinib for 2hr followed by stimulation with OSM for 20 min. The representative data of three independent experiments is shown.

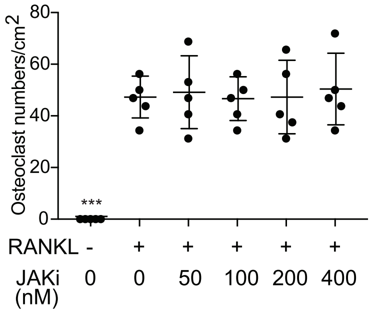

Fig. S8. No effect of the JAK inhibitor on osteoclat precursor cells in osteoclastogenesis. All data are expressed as the mean ± SD. ***p <0.001 by one-way ANOVA with the Holm-Sidak test vs. all other groups.

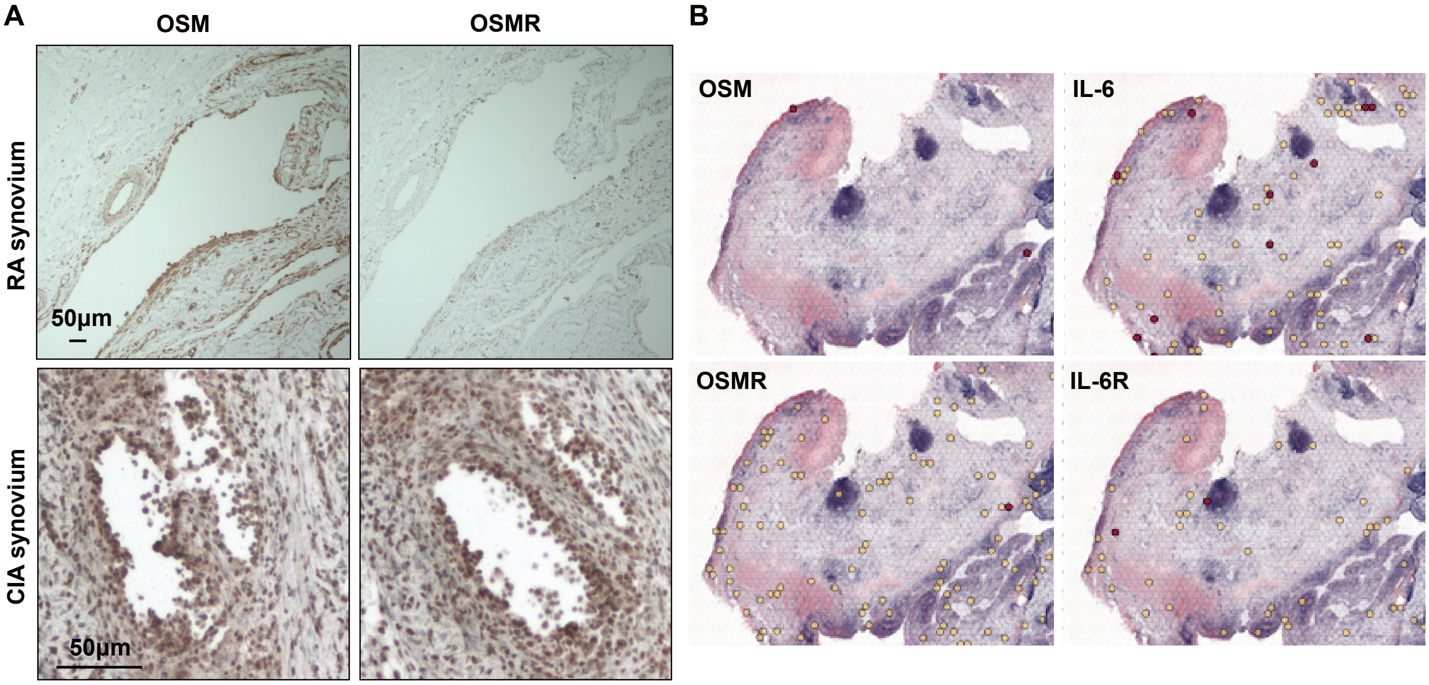

Fig. S9. Statial expression of OSM, OSMR, IL-6 and IL-6R in the arthritic synovium

(A) Immunohistochemistry of RA (upper) and CIA (lower) synovium. Expression of OSM and OSMR in RA (upper) and CIA (lower) synovium. The representative data. Scale bar 50μm.

(B) Spatial transcriptomics (https://www.immport.org/shared/study/SDY2213) of RA synovium. Expression of OSM, IL-6, OSMR, and IL-6R on the spatial transcriptomics slide is shown.
